# Essential role for PfHSP40 in asexual replication and thermotolerance of malaria parasites

**DOI:** 10.1371/journal.ppat.1013313

**Published:** 2025-07-08

**Authors:** Brianne Roper, Deepika Kannan, Emily S. Mathews, Audrey R. Odom John

**Affiliations:** 1 Division of Infectious Diseases, Children’s Hospital of Philadelphia, Philadelphia, Pennsylvania, United States of America; 2 Perelman School of Medicine, University of Pennsylvania, Philadelphia, Pennsylvania, United States of America; 3 Department of Pediatrics, Washington University School of Medicine, St. Louis, Missouri, United States of America; Johns Hopkins Bloomberg School of Public Health, UNITED STATES OF AMERICA

## Abstract

*Plasmodium falciparum,* the parasite responsible for nearly all cases of severe malaria, must survive challenging environments to persist in its human host. Symptomatic malaria is characterized by periodic fevers corresponding to the 48-hour asexual reproduction of *P. falciparum* in red blood cells. As a result, *P. falciparum* has evolved a diverse collection of heat shock proteins to mitigate the stresses induced by temperature shifts. Among the assortment of heat shock proteins in *P. falciparum*, there is only one predicted canonical cytosolic J-domain protein, PfHSP40 (PF3D7_1437900). Here, we generate a PfHSP40 tunable knockdown strain of *P. falciparum* to investigate the biological function of PfHSP40 during the intraerythrocytic lifecycle. We determine that PfHSP40 is required for malaria parasite asexual replication and survival of febrile temperatures. Previous reports have connected proteotoxic and thermal stress responses in malaria parasites. However, we find PfHSP40 has a specific role in heat shock survival and is not essential for mitigating the proteotoxic stresses induced by artemisinin or proteosome inhibition. Following PfHSP40 knockdown, malaria parasites have a cell cycle progression defect and reduced nuclear replication. Untargeted proteomics reveal PfHSP40 depletion leads to a multifaceted downregulation of DNA replication and repair pathways. Additionally, we find PfHSP40 knockdown sensitizes parasites to DNA replication inhibition. Overall, these studies define the specialized role of the J-domain protein PfHSP40 in malaria parasites during the blood stages of infection.

## Author summary

Malaria parasites have evolved to survive and cause disease in humans even when they are challenged by the temperature shifts of fever. We are interested in uncovering how malaria parasites persist despite exposure to febrile temperatures. We know the parasite has a diverse collection of heat shock proteins that are important for proper folding of many proteins within cells during stress conditions. In this study, we define the role of one specific heat shock protein, PfHSP40, by generating a strain of the human malaria parasite *P. falciparum* where we control the expression of PfHSP40 during the red blood cell stages of infection. We find that PfHSP40 is required for malaria parasites to replicate in red blood cells. We demonstrate PfHSP40 expression to be vital for malaria parasite survival of febrile temperature stress. Additionally, we determine that without PfHSP40, DNA replication and repair is disrupted. Our work has uncovered essential parasite biology that may be exploited for the development of new antimalarials.

## Introduction

Malaria is a persistent global health threat, causing over six hundred thousand deaths annually [1]. *Plasmodium* spp. are the intracellular parasites responsible for malaria and *Plasmodium falciparum* is the deadliest of all human malaria species. Humans respond to *P. falciparum* infection with a cyclical fever response corresponding to the synchronized replication of parasites in red blood cells [2–4]. As a result, *P. falciparum* has evolved unique mechanisms to tolerate host-induced febrile temperatures [5–9]. Resistance to front-line artemisinin-based combination therapies and other antimalarial compounds impedes efforts to the treat malaria worldwide [1]. Recent work has shown mechanisms the parasite employs to protect itself from febrile temperatures also appear to be utilized by the parasite to survive artemisinin treatment [9,10]. Defining fundamental malaria parasite biology as it pertains to stress survival is crucial to inform antimalarial design and combat rising drug resistance.

Processes related to the apicoplast in *P. falciparum* have been shown to be involved in the ability of malaria parasites to survive febrile temperatures [9,11]. The apicoplast houses the MEP pathway which generates isopentyl pyrophosphate (IPP), the building block for large isoprenoids that post-translationally modify proteins via prenylation [12]. Our previous work has shown that protein prenylation, specifically farnesylation, is immediately required for *P. falciparum* to survive temperature shifts like those experienced by the parasite during fever [11]. *P. falciparum* has a modest set of only four farnesylated proteins—one of these is the canonical J-domain protein PfHSP40 (PF3D7_1437900), which has yet to be functionally characterized for its biological role in parasite growth and thermotolerance [13,14].

Molecular chaperones are crucial for folding nascent peptides and maintaining protein integrity during stress conditions [15]. To maintain protein homeostasis, molecular chaperones work in a complex network of co-chaperoning interactions [16]. J-domain proteins are a class of molecular chaperone with essential housekeeping and stress response functions. The role of J-domain proteins is to load misfolded peptide substrates onto HSP70 chaperones and mediate protein refolding by stimulating the ATPase activity of HSP70 [17]. The diverse collection of J-domain proteins guide substrate specificity for the restricted number of encoded HSP70 chaperones [18–20]. *P. falciparum* has an expanded set of J-domain proteins indicating the parasite has specifically evolved this class of chaperones for surviving the unique facets of the lifecycle [8,21,22]. In *P. falciparum,* PfHSP70-1 (PF3D7_0818900) partners with the J-domain protein PfHSP40 and is required for parasite heat shock recovery; however, the role of PfHSP40 in thermotolerance remains undetermined [11,23–25].

In this study, we use a conditional knockdown approach to investigate the biological role of PfHSP40 in *P. falciparum* asexual replication, sensitivity to artemisinin, and thermotolerance. We find PfHSP40 is an essential protein for replication of malaria parasites in red blood cells and is vital for heat shock recovery. Additionally, we determine PfHSP40 is not required for mitigating the proteotoxic stresses induced by artemisinin or proteosome inhibition. Interestingly, we find PfHSP40 depletion is associated with a multifaceted downregulation of DNA replication and repair pathways as well as an increased sensitivity to DNA replication inhibition. Altogether, this work teases apart the specialized role of PfHSP40 and uncovers unique biology as it pertains to thermotolerance and DNA replication in malaria parasites.

## Results

### PfHSP40 is an essential protein for *P. falciparum* asexual replication

**Fig 1:**
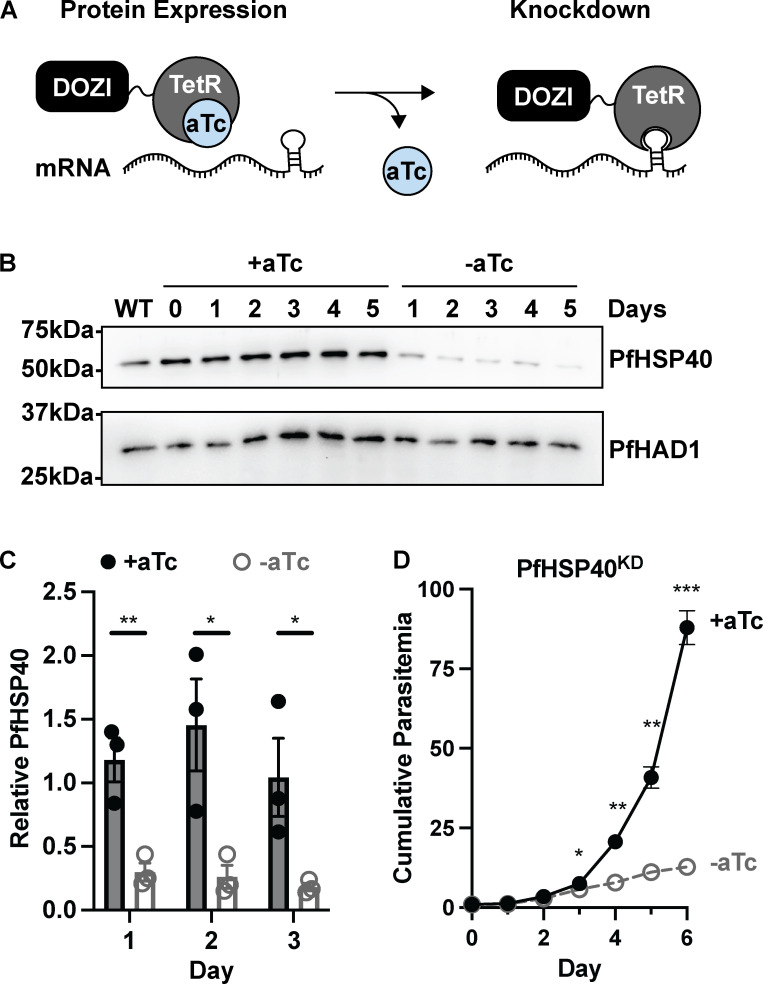
PfHSP40 expression is essential for *P. falciparum* replication. **A)** The TetR-DOZI system controls protein expression. Adding hairpin aptamers to the mRNA of the gene of interest allows for knockdown by removal of anhydrotetracycline (aTc) from culture media. **B)** Representative anti-PfHSP40 immunoblots of parasite lysates collected from PfHSP40^KD^ parasites + /- aTc for 5 days. HAD1 was used as a loading control. Without aTc, PfHSP40 expression decreases starting at 24hrs. Blot is representative of 3 biological replicates. **C)** Quantification of the relative expression level of PfHSP40 to loading control PfHAD1 from PfHSP40 immunoblots + /- aTc on days 1-3. Data represents the mean + /- SEM of 3 biological replicates, parametric unpaired t-tests were performed (*p < 0.05, **p < 0.01). **D)** Growth assay of asynchronous PfHSP40^KD^ parasites. Cumulative parasitemia (percentage infected erythrocytes) was quantified by flow cytometry every 24hrs from cultures grown + /- aTc. Parasites were split 1:6 after day 4. Data represents the mean + /- SEM of 3 biological replicates, missing error bars are too small to be visualized. Parametric unpaired t-tests were performed (*p < 0.05, **p < 0.01, ***p < 0.001).

Forward genetic screens and the inability to generate a PfHSP40 knockout strain suggest PfHSP40 is an essential gene in blood-stage *P. falciparum* [11,26]. Therefore, to investigate the biological function of PfHSP40 in asexual parasites, we employed the TetR-DOZI conditional knockdown system to control expression of PfHSP40 [27]. Due to regulation of the TetR-DOZI fusion protein and hairpin aptamers added to the mRNA of PfHSP40, PfHSP40 is expressed when anhydrotetracycline (aTc) is added to the culture media but substantially reduced when aTc is removed (Fig 1A). Using a CRISPR/Cas9 genomic editing strategy, the native locus of PfHSP40 was replaced with necessary components for TetR-DOZI regulation and confirmed by PCR amplification from genomic DNA (S1A Fig). Upon removal of aTc, immunoblotting parasite lysates of the TetR-DOZI regulated PfHSP40 knockdown strain (hereafter referred to as PfHSP40^KD^) show reduced PfHSP40 expression beginning one day without aTc, confirming that TetR-DOZI regulates PfHSP40 as expected (Fig 1B and 1C).

To evaluate whether PfHSP40 plays a role in asexual replication of *P falciparum*, we quantified asynchronous parasite growth over time after PfHSP40 knockdown. We find that PfHSP40 expression is essential for asexual replication of malaria parasites (Fig 1D). Interestingly, reduced PfHSP40 expression did not lead to a significant growth difference until after the first full 48-hour cycle of replication. We next examined whether the PfHSP40 knockdown effect was irreversible by adding aTc back to parasite cultures after two or four days without aTc (S1B and S1C Fig). Adding aTc back to the cultures after two days without aTc, despite substantial reduction of PfHSP40 expression, parasites continue to grow with normal kinetics. Replenishing aTc after four days without aTc, when significant parasite growth delay has already occurred, parasites begin to replicate again. These results demonstrate that PfHSP40 knockdown substantially reduces parasite replication potentially due to disturbing without triggering irreversible cell death. This non-lethal effect could result from the lingering presence of PfHSP40 molecules in our knockdown or a compensatory role of other J-domain proteins in the parasite.

### PfHSP40 knockdown sensitizes *P. falciparum* to heat shock

**Fig 2:**
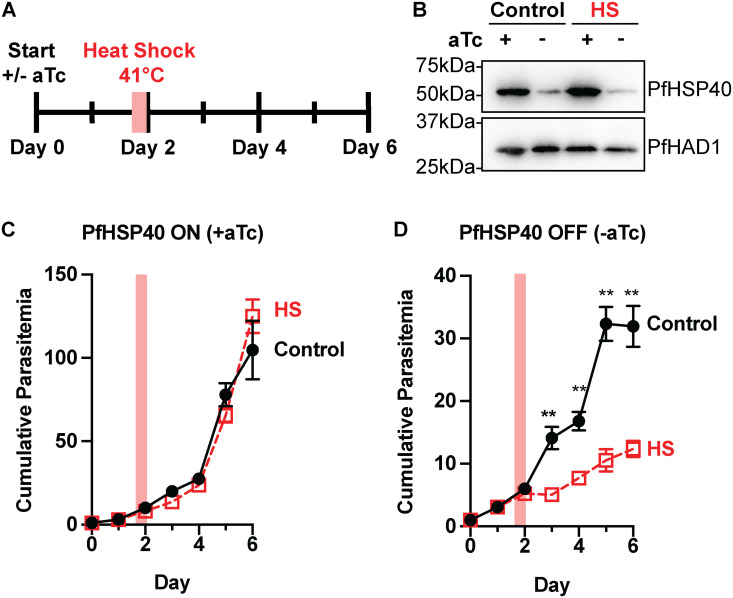
PfHSP40 expression is vital for *P. falciparum* survival following heat stress. **A)** Experimental design to assay thermotolerance: PfHSP40^KD^ parasites were subjected to a 6hr 41°C heat shock (HS) on day 2 + /- aTc. **B)** Anti-PfHSP40 immunoblot of PfHSP40^KD^ parasites + /- aTc in the control or HS condition collected immediately following HS. Blot is representative of 3 biological replicates. Parasitemia was measured by flow cytometry collecting every 24hrs following the HS for **C)** HSP40 expression on (+aTc) or D) off (-aTc). Cultures were split 1:6 after day 4 collection. Data represents the mean + /-SEM of 3 biological replicates, missing error bars are too small to visualize. Parametric unpaired t-tests were performed (**p < 0.01).

PfHSP40 is one of only four proteins that are modified post-translationally by farnesylation in *P. falciparum* [13,14]. Previous work has shown that protein farnesylation is required for malaria parasite thermotolerance [11]. We were able to take advantage of the delayed growth defect phenotype of the PfHSP40^KD^ parasites to determine if PfHSP40 plays a role in parasite heat shock recovery. Monitoring parasite growth after a 6-hour 41°C heat shock during the second day of PfHSP40 knockdown, we find that PfHSP40 depletion sensitizes parasites to heat stress (Fig 2). A similar but more modest effect was observed by heat shock on the first day of PfHSP40 knockdown, possibly mitigated by residual PfHSP40 (S2 Fig). These data demonstrate that PfHSP40 is essential for malaria parasites to survive fever-relevant heat shock temperatures.

### The PfHSP40 phenotype is partially rescued by growth at lower temperatures

**Fig 3:**
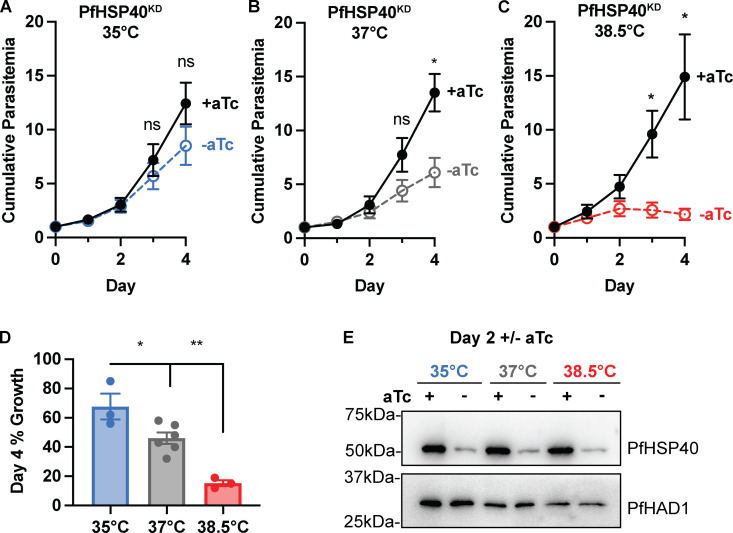
The PfHSP40 phenotype is partially rescued at lower temperatures. Growth assay of asynchronous PfHSP40^KD^ parasites measuring parasitemia by flow cytometry every 24hrs cultured + /- aTc at A) 35°C B) 37°C and C) 38.5°C. Data represents the mean + /- SEM of 3 biological replicates. **D)** Normalizing the cumulative parasitemia on day 4 to the + aTc condition shows the PfHSP40 knockdown effect becomes more pronounced as temperature increases. Data represents the mean + /- SEM of biological replicates. Parametric unpaired t-tests were performed (*p < 0.05, **p < 0.01). **E)** Anti-PfHSP40 immunoblots of PfHSP40^KD^ parasites + /- aTc at the indicated temperatures collected on day 2 + /-aTc. Blot is representative of 3 biological replicates.

While the temporary heat exposure showed PfHSP40 plays a role in heat shock recovery, we sought to evaluate whether PfHSP40 knockdown affects growth under constant exposure to different temperature conditions. Using a range of temperatures from 35°C to 38.5°C, we find that the requirement of PfHSP40 for parasite replication is temperature dependent (Fig 3). Growing parasites at 38.5°C, PfHSP40 knockdown leads to reduced parasitemia earlier than what was observed at 37°C (Fig 3C). Interestingly, at 35°C parasite replication does not show a significant reduction with PfHSP40 knockdown (Fig 3A). These results seemingly show a dose-effect, such that reduced PfHSP40 expression has a greater inhibitory effect on parasite replication with increasing temperature (Fig 3D). This demonstrates that not only is PfHSP40 expression critical for survival of fever-relevant heat pulses, but PfHSP40 also is an essential regulator of parasite growth under constant elevated temperature.

### PfHSP40 is not essential for mitigating stresses from artemisinin or proteosome inhibition

**Fig 4:**
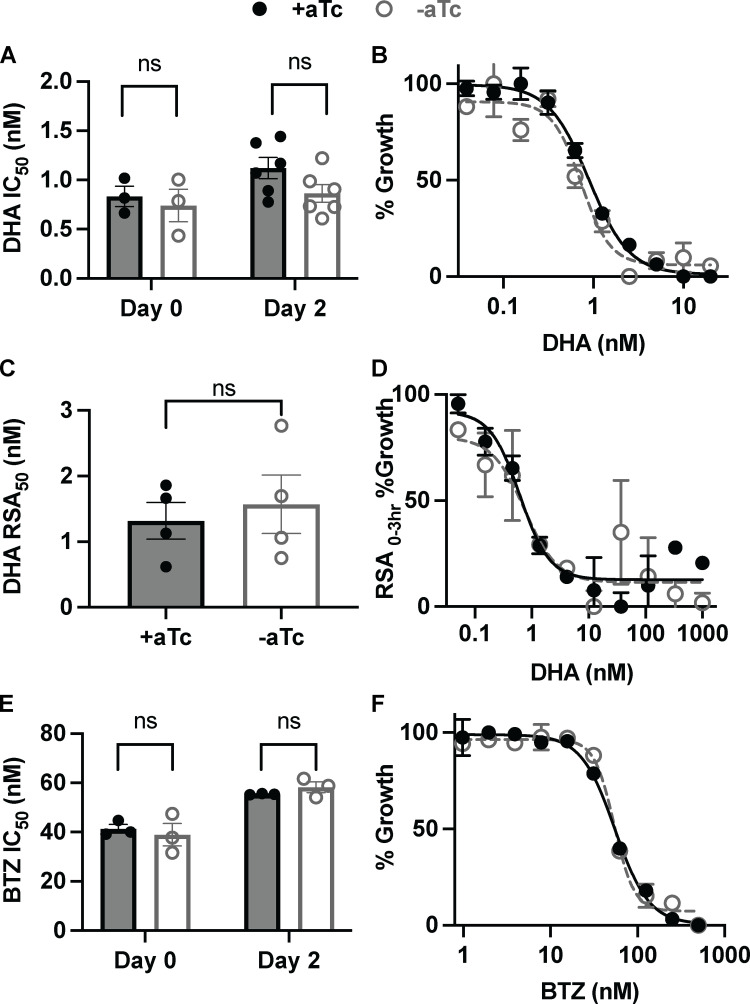
PfHSP40 is not essential for mediating survival to chemically induced proteotoxic stress. **A)** Half-maximal inhibitory concentration (IC_50_) values of 72hr dose-response curves started at day 0 or day 2 + /-aTc of asynchronous PfHSP40^KD^ parasites with dihydroartemisinin (DHA). **B)** Representative IC_50_ curve of day 2 + /- aTc DHA treatment. **C)** DHA concentration value resulting in 50% growth inhibition during Ring-Stage Survival Assays (RSA_50_). A 6hr DHA pulse at varying concentrations was given to 0-3hr ring stage parasites on day 2 + /- aTc, parasite growth measured after 72hrs. **D)** Representative RSA_50_ curve. **E)** IC_50_ summary values of 72hr dose-response curves started at day 0 or day 2 + /-aTc of asynchronous PfHSP40^KD^ parasites with proteosome inhibitor Bortezomib (BTZ). **F)** Representative IC_50_ curve of day 2 + /- aTc BTZ treatment. A, C, and E summary values are of biological replicates performed in technical duplicate, data represents the mean + /-SEM. E, D, and F are representative dose-response curves for 1 of the biological replicates showing the mean + /- SEM of the technical duplicates. No significance was found for the summary values performing parametric unpaired t-tests.

The antimalarial mechanism of artemisinin is, in part, due to an accumulation of damaged proteins [28]. Mechanisms of protection utilized by malaria parasites in heat shock recovery have been harnessed by the parasite to survive treatment with artemisinin [9,10]. Therefore, we measured sensitivity to the artemisinin derivative dihydroartemisinin (DHA) in PfHSP40^KD^ parasites + /-aTc to determine whether PfHSP40 mitigates proteotoxic stresses induced by this inhibitor. Even after two days of PfHSP40 depletion, we found no increased sensitivity to DHA. This phenotype was observed both under constant DHA treatment in asynchronous parasites (Fig 4A and 4B) as well as for a 6-hour DHA pulse against 0–3 hour ring stage parasites (RSA_0–3_; Fig 4C and 4D). To determine if phenotype was specific to DHA or applicable to other forms of chemically induced proteotoxic stress, we tested PfHSP40^KD^ sensitivity to bortezomib (BTZ), a potent proteosome inhibitor, with similar results (Fig 4E and 4F) [29]. Our findings highlight that the cellular response to temperature and proteotoxic stresses have distinct features. PfHSP40 has a specific role in heat stress survival and is not essential for mitigating the proteotoxic stresses induced by artemisinin or proteosome inhibition.

### PfHSP40 knockdown leads to a cell cycle progression defect and reduced nuclei replication

**Fig 5:**
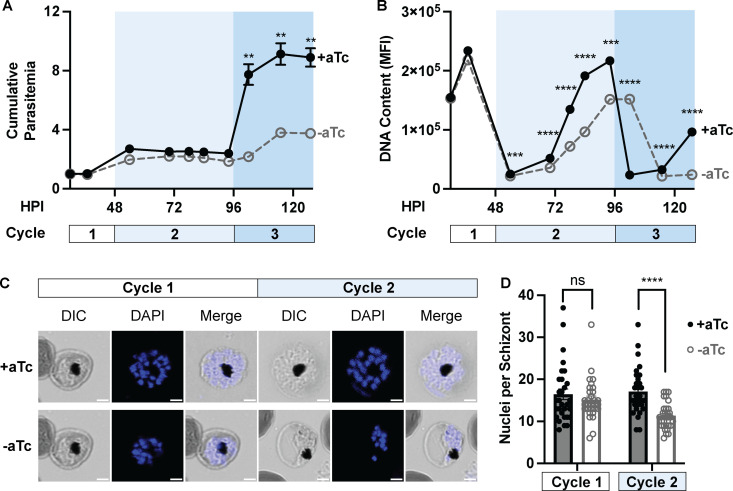
PfHSP40 depletion leads to a delayed reduction in parasite DNA content and nuclei generation. Tightly synchronized PfHSP40^KD^ parasites were starting + /-aTc at 8hrs post invasion in (HPI) and monitored through the third cycle of replication. A) Calculating cumulative parasitemia via flow cytometry shows loss in parasitemia entering the third cycle of PfHSP40 knockdown (-aTc). B) The median fluorescence intensity (MFI) of the DNA content of infected erythrocytes measured by flow cytometry demonstrates PfHSP40 knockdown corresponds to reduced level of DNA content throughout cycle 2. Additionally, the peak in DNA content is approximately 8hrs behind the + aTc condition. Data represents the mean + /- SEM of 3 biological replicates, missing error bars are too small to visualize. Parametric, unpaired t-test were performed (**p < 0.01, ***p < 0.001, ****p < 0.0001). C) Representative image of the DAPI stained segmented schizonts used to quantify nuclei in the first and second cycle of PfHSP40 knockdown, scale bar represents 2 microns. D) Nuclei of segmented schizonts + /- aTc were counted for 30 cells across 3 biological replicates in the first two replication cycles. Parametric, unpaired t-test were performed (****p < 0.0001).

Having established PfHSP40 as an essential protein for parasite growth and thermotolerance, we next pursued deeper understanding of the mechanism by which decreased PfHSP40 expression leads to reduced parasite replication in red blood cells. We monitored lifecycle progression in tightly synchronized PfHSP40^KD^ parasites with and without aTc by microscopy as well as flow cytometry (Fig 5). Measuring cumulative parasitemia, we find that there is no difference in parasitemia until entering the third replication cycle (Fig 5A). Examining lifecycle progression by light microscopy revealed a developmental lag during the second cycle, beginning during parasite schizogony (S3A Fig). In the second cycle, the PfHSP40 knockdown (-aTc) condition slows during late schizogony, eventually entering the third cycle, approximately 8 hours behind and with fewer parasites. The DNA content of these cells (measured by flow cytometry) highlights the lag in parasite development during the second cycle, as the peak in DNA content -aTc is approximately 8 hours behind the + aTc condition (Fig 5B). Additionally, throughout the entire second cycle the PfHSP40 knockdown parasites have reduced DNA content (Fig 5B).

To evaluate whether these findings represented a defect in nuclear replication, we quantified the number of nuclei in segmented schizonts during the first two cycles of PfHSP40 knockdown. We find that PfHSP40 knockdown corresponded to a reduced number of nuclei per schizont in the second replication cycle (Fig 5C and 5D). Altogether these results show that PfHSP40 depletion leads to a delayed cell cycle progression defect during the trophozoite to schizont transition, reduced DNA content, and nuclear replication deficiency, ultimately resulting in fewer parasites.

### Downregulation of DNA replication and repair proteins following PfHSP40 depletion

**Fig 6:**
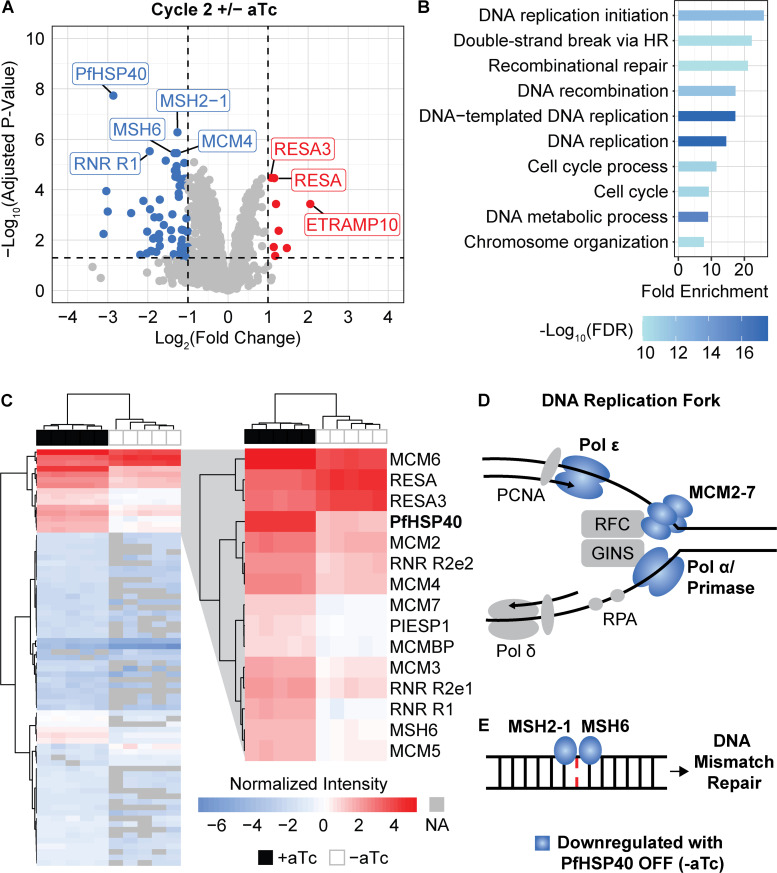
Proteomics reveal downregulation of DNA replication and repair pathways upon PfHSP40 knockdown. Synchronized PfHSP40^KD^ cultures were started + /- aTc as rings and collected for proteomics as trophozoites in cycles 1 and 2 of knockdown for 5 biological replicates. **A)** Volcano plot of cycle 2 + /- aTc differential abundance analysis, downregulated genes in blue, upregulated in red. Differential abundance analysis was performed comparing + /- aTc for each cycle using LIMMA with empirical Bayes smoothing and Benjamini-Hochberg method for multiple test corrections. Significant hits had an adjusted P-value < 0.05 and absolute log_2_(fold change) > 1. **B)** Biological Process Gene Ontology of the 67 downregulated proteins using Shiny GO 8.0. **C)** Heat map of the normalized intensity of all 75 differentially expressed proteins in cycle 2 + /- aTc across 5 biological replicates detected by proteomics. Hierarchical clustering was performed using Euclidean distance and Ward method for columns and rows. Peptides that were not detected are NA in grey. The top 3 clusters which include PfHSP40 are zoomed in. Fully annotated heatmap shown in S4 Fig. **D)** Of the predicted proteins present at the DNA replication fork of *P. falciparum* during asexual replication, in cycle 2 of PfHSP40^KD^ -aTc we detect reduced abundances of the full MCM complex (MCM2-7), DNA polymerase alpha (Polα) subunit B, DNA primase large and small subunits, and DNA polymerase epsilon (Polε) subunit **B. E)** MSH2-1 and MSH6 detect DNA mismatches at the beginning of the mismatch repair pathway in *P. falciparum* and both proteins have reduced abundance in cycle 2 of PfHSP40 knockdown.

Because PfHSP40 is predicted to have a role in maintaining protein homeostasis, we hypothesized that the nuclear replication defect we observed might be due to changes in intracellular parasite protein expression during PfHSP40 knockdown. For this reason, we performed whole-cell proteomics on isolated trophozoites in cycle one and two of PfHSP40 knockdown [11,24,25]. Consistent with the lack of phenotype in the first cycle + /- aTc, PfHSP40 was the only protein with significantly different expression at this time point (S4 Fig and S1 Table). This reduction in the abundance of PfHSP40 demonstrates that the TetR-DOZI regulation of PfHSP40 begins within the first cycle of aTc removal, but there are yet to be notable changes of any other protein within these parasites. In contrast, in the second cycle of PfHSP40 depletion we detected a total of 75 proteins with significantly different abundances, 67 downregulated and 8 upregulated proteins (Fig 6A and S2 Table). PfHSP40 is the most significantly downregulated protein in the second cycle of PfHSP40 knockdown. Performing hierarchical clustering of the 75 hits revealed 14 other proteins regulated similarly to PfHSP40 across our 5 biological replicates (Fig 6C).

Among the upregulated proteins are RESA (ring-infected erythrocyte surface antigen protein) and RESA3, J-domain proteins that are exported to the host red blood cell [22]. Interestingly, RESA has been shown to be involved in protection of the red blood cell membrane during heat shock [30]. These results suggest that with loss of PfHSP40 there is an upregulation of other J-domain proteins; however, based on our heat shock data, this upregulation appears insufficient for parasite thermotolerance in the absence of PfHSP40. Interestingly, the known PfHSP40 co-chaperone, PfHSP70-1, was not differentially expressed in the first two replication cycles, indicating regulation of PfHSP70-1 levels appears to be independent of PfHSP40 expression. An important limitation of our proteomics screen was the use of parasites isolated from red blood cells. For this reason, our study does not capture any significant changes in exported proteins, including exported molecular chaperones, that may contribute to our observed phenotypes.

Gene ontology analysis of the 67 downregulated genes reveals that proteins in DNA replication and repair pathways are affected by PfHSP40 knockdown (Fig 6B and S3 Table). We detect reduced expression of all 6 of the mini chromosome maintenance proteins (MCM 2-7). MCM2-7 drive formation of DNA prereplication complexes as part of DNA replication licensing and comprise the helicase involved in DNA unwinding during replication (Fig 6D) [31,32]. In addition to the MCM complex, we detect downregulation of the DNA polymerase alpha (Polα) subunit B (PF3D7_1463300), DNA primase large (PF3D7_1438700) and small subunits (PF3D7_0910900), and DNA polymerase epsilon (Polε) subunit B (PF3D7_1234300), which comprise many of the known components involved in DNA replication forks in *P. falciparum* (Fig 6D) [33,34]. Additionally, we see reduced levels of MSH2-1 and MSH6, which work together to detect DNA mismatches in the initial steps of the mismatch repair pathway of *P. falciparum* (Fig 6E) [35]. These proteomics results demonstrate that PfHSP40 is required for homeostasis of DNA replication and repair machinery in malaria parasites.

### PfHSP40^KD^ parasites are hypersensitized to inhibition of DNA replication

**Fig 7:**
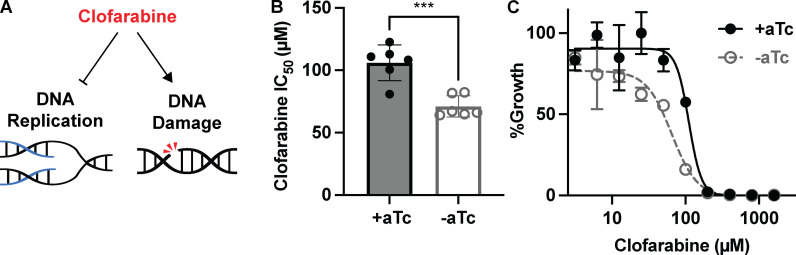
PfHSP40 knockdown sensitizes parasites to DNA replication inhibition. **A)** Clofarabine is a nucleoside analog which inhibits DNA replication while simultaneously eliciting DNA damage. **B)** IC_50_ summary values of 96hr dose-response curves started with synchronous PfHSP40^KD^ ring parasites with Clofarabine. Data represents the mean + /-SEM of 6 biological replicates performed in technical duplicate. Parametric unpaired t-tests were performed (***p < 0.001). **C)** Representative IC_50_ curve 96hr Clofarabine treatment for 1 of the biological replicates showing the mean + /- SEM of technical duplicates.

Our proteomics indicated that DNA replication and repair pathways are markedly disrupted in during PfHSP40 knockdown. Clofarabine is a nucleoside analog drug that inhibits DNA replication machinery such as polymerases and primases, while simultaneously inducing DNA damage (Fig 7A) [36,37]. We find that PfHSP40 knockdown corresponded with increased sensitivity to clofarabine, indicating PfHSP40^KD^ parasites are hypersensitized to DNA replication inhibition (Fig 7B and 7C). This functionally validates our proteomics findings and highlights the dysregulation of DNA replication and repair during PfHSP40 knockdown.

## Discussion

Replication of the malaria parasite within its human host requires the ability to tolerate modest temperature shifts, especially febrile temperatures >38.0°C. Understanding malaria heat shock survival can define essential parasite biology and elucidate requirements for pathogenesis. The temperature changes during the malaria parasite lifecycle, combined with an unusually aggregation-prone proteome, have raised questions in the field as to how *P. falciparum* maintains protein homeostasis despite these challenges [38–40]. Molecular chaperones comprise a diverse class of proteins utilized in all biological systems to maintain protein integrity under standard and various stress conditions, including temperature stress. *P. falciparum* is thought to have a uniquely specialized collection of heat shock proteins that allow for protein stability in a temperature shifting environment [20,41]. In this work, we establish that the molecular chaperone, PfHSP40, is an essential regulator of *P. falciparum* blood stage replication and thermotolerance.

PfHSP40 belongs to the J-domain protein family that facilitate protein refolding by delivering misfolded peptides substrates to HSP70 chaperones and stimulating HSP70 ATPase activity [17]. The restricted number of HSP70s in *P. falciparum* have their substrate specificity dictated by the wide assortment of J-domain proteins [18–20]. PfHSP40 is classified as the canonical, cytosolic J-domain protein and a fraction of PfHSP40 molecules are post-translationally modified by the isoprenyl group farnesyl [13,14,21]. Our previous work demonstrated that farnesylation is required for *P. falciparum* thermotolerance, and PfHSP40 is the sole farnesylated protein in *P. falciparum* with predicted roles in heat shock survival [11,13,14]. Interestingly, farnesylation of the PfHSP40 homolog in yeast and plants has been shown to be required for organismal thermotolerance, suggesting an evolutionarily conserved role [42–44]. Here we provided evidence that PfHSP40 has a specialized temperature-related essential function in malaria parasites. We find PfHSP40 is required for asexual parasite growth during both constant and temporary elevated temperature exposure; however, parasite growth is less dependent on PfHSP40 expression at lower temperatures. These results seemingly showed a dose-effect, such that reduced expression of PfHSP40 was more detrimental with increasing temperatures, highlighting the specificity in the essential function of PfHSP40 for heat stress survival.

Multiple studies have broadly connected intracellular processes that are essential for heat shock survival to parasite survival under treatment with the front-line antimalarial artemisinin. For example, heat stress and artemisinin induce similar transcriptional programs, including changes in expression of exported proteins and proteins involved in the unfolded protein response and lipid metabolism [45]. Heat-sensitive *P. falciparum* mutants tend to be sensitive to artemisinin and the proteosome inhibitor bortezomib [9]. Pre-treating malaria parasites with heat reduces their susceptibility to artemisinin, strongly connecting protective cellular processes under both stresses [10]. For these reasons, we were surprised to find that, although PfHSP40 is required for thermotolerance, it does not appear to be essential for mitigating the proteotoxic stresses caused by artemisinin derivative, dihydroartemisinin, or proteosome inhibitor, bortezomib. This could be due to distinct intracellular dysfunctions evoked by these stresses, requiring particular responses. Alternatively, these results may suggest that other J-domain proteins play a compensatory role during PfHSP40 knockdown and thus mitigate artemisinin or bortezomib proteotoxic stresses.

In *P. falciparum*, the specialized function of PfHSP40 appears to be related to homeostasis of DNA replication and repair machinery. During PfHSP40 knockdown parasites show a developmental lag during the DNA replication phase of the lifecycle, have a reduction in DNA content per cell and the number of nuclei per schizont. Our proteomics overwhelmingly point to DNA replication and repair pathways being downregulated upon PfHSP40 depletion. Finally, we uncovered increased sensitivity to clofarabine which inhibits DNA replication and also elicits DNA damage. PfHSP40-mediated thermotolerance may be linked to its role in regulating the homeostasis of the DNA repair machinery. Malaria parasites upregulate DNA damage repair pathways and have detectable double stranded DNA breaks during heat shock [8,9,46]. The PfHSP40 homolog in yeast has been shown to be essential in regulation of DNA synthesis and damage responses; however, the strategy by which PfHSP40 mitigates DNA replication and repair is unclear in *P. falciparum* [47]. DNA replication and repair downregulation during PfHSP40 knockdown suggests expression of these DNA protein components has a common mechanism of regulation. Understanding the mechanism by which reduced expression of PfHSP40 leads to disruption of DNA replication and repair machinery will likely yield additional insights into regulation of protein expression in *P. falciparum*.

In summary, this work defines the essential function of the J-domain protein, PfHSP40, in *P. falciparum* asexual replication and thermotolerance. Our data tease apart the specialized role of PfHSP40, highlighting unique mechanisms malaria parasites have evolved to survive under different stress conditions. We identify PfHSP40 as a potential regulator of DNA replication and repair pathways, establishing a foundation for future inquiries in nuclear replication dynamics. Finally, this study elucidates vital parasite biology which could be exploited in the development on novel antimalarials and contributes to the broader understanding of this unique subclass of J-domain molecular chaperones.

## Materials and methods

### Parasite strains and cultures

Parasites were cultured in RPMI medium (Gibco) with the addition of 27mM NaHCO_3_, 11mM glucose, 5mM HEPES, 0.01mM thymidine, 1mM sodium pyruvate, 0.37mM hypoxanthine, 10ug/ml gentamicin, and 5g/L Albumax (Thermo Fisher Scientific) and maintained at 37°C in 5% O_2_, 5% CO_2_, 90% N_2_ in a 2% suspension of human red blood cells. The wild-type strain 3D7 (MRA-102) was obtained from BEI Resources Repository, NIAID, NIH. Deidentified red blood cells of either A + , AB + , or O+ blood type were obtained from the Children’s Hospital of Philadelphia Blood Bank and BioIVT. Parasites were synchronized using a combination of 5% sorbitol (Sigma: S889) and Percoll gradients (Sigma: P4927).

The PfHSP40 regulatable knockdown strain, PfHSP40^KD^, was generated by employing CRISPR/Cas9 to edit the native locus of PfHSP40 (Pf3D7_1437900) and incorporate TetR-DOZI regulation as previously described [48,49]. The pSN054 linear plasmid contained the segments that replaced the native locus via homologous recombination. PCR amplification of the PfHSP40 5’UTR (primers A + B) and 3’UTR (primers C + D) from genomic DNA of 3D7 *P. falciparum* was done using Primestar GXL DNA Polymerase (Takara: R050A) (S4 Table). Fragments were inserted into the Fse-I and I-SceI restriction sites in the pSN054 vector using the NEBuilder HIFI assembly master mix (NEB: E2621) and transformed into BigEasy Electocompentant Cells (Lucigen: 60224). The coding sequence of PfHSP40 with an N-terminal HA tag along with silent mutations to remove gRNA cut sites was ordered from Genewiz (S1 Appendix), PCR amplified (primers E + F) and inserted into the pSN054 PfHSP40 5’ and 3’ UTR plasmid at the AsiSI/BsiWI cut sites (S4 Table). The pAIO3 vector contained the Cas9 enzyme along with the guide RNA (sequence GTATGTGTGTATTGAAAACA) which was inserted at the AflII cut site using TOPO cloning (Invitrogen: K2800-20SC) according to the manufacturer’s protocol. Plasmid sequences were confirmed via Sanger sequencing.

For transfection, 50μg of each plasmid was precipitated and resuspended in 400μL of Cytomix (120mM KCl, 0.15mM CaCl_2_, 2mM EGTA, 5mM MgCl_2_, 10mM K_2_HPO_4_, 25mM HEPES, pH 7.6). Synchronized 3D7 ring parasites at roughly 5% parasitemia were washed with Cytomix and resuspended in the 400μL Cytomix-DNA mixture. Cells were electroporated at 950μF capacitance and 0.31kV using a Biorad Genepulser Xcell. Cells were washed with media and cultured immediately with the addition of 50-100nM aTc (Caymen Chemicals: 10009542) diluted in DMSO. Starting 24 hours post-transfection, parasites were selected for using 2.5 μg/mL blasticidin (Invitrogen: R210-01).

From the pooled population of transfected parasites, individual clonal populations were grown out. Five individual clones were isolated and their growth + /- aTc was characterized to have a similar phenotype. One of the clones was used for experiments in biological replicates, defined as cultures grown and treated separately for at least 1 week.

Genomic integration of the TetR-DOZI cassette was confirmed using test PCR as well as sequencing PCR fragments from chloroform extracted genomic DNA from PfHSP40^KD^ clones amplified with primer pairs A + F and G + H (S4 Table). Sequencing confirmed genomic integration occurred within the PfHSP40 second exon and included aptamers, the TetR-DOZI cassette, and blasticidin selection marker between the 3’ end of the PfHSP40 sequence and the beginning of the PfHSP40 3’UTR.

### Western blotting

Parasite lysates were obtained by 1% saponin lysing 25mls of parasite cultures followed by cold PBS washes. Samples were stored in -80°C until ready to sonicate. All steps for sample preparation were performed at 4°C. Samples were washed in lysis buffer (10% glycerol, 100mM NaCl, 100mM Tris pH 7.5, 1mM MgCl_2_, 1mM DTT, with an EDTA-free protease inhibitor cocktail mini-tablet (Roche: 11836170001)) twice. Parasite pellets were resuspended in lysis buffer then sonicated with 6 cycles, 10 second pulses at 40% amplitude with a FisherBrand Model 120 Sonic Dismembrator. After sonication, samples were stored at -80°C. For western blotting, sonicated supernatant was diluted in SDS-PAGE buffer with 2-β-mercaptoethanol and boiled for 10min. The equivalent of approximately 1x10^7^ parasites were loaded on 12% SDS-PAGE gels and ran at 120V, transferred to a PVDF membrane using the BioRad Transblot Turbo system with TBT-0.05% SDS buffer, and blocked overnight with 5% BSA in PBS_T_ rocking at 4°C. Primary antibody and secondary antibodies were incubated for an hour at room temperature rocking with 3 10-minute PBS_T_ washes in between.

*P. falciparum* PfHSP40 rabbit anti-sera generated previously [11] was used at a 1:5000 dilution, *P. falciparum* HAD1 rabbit anti-sera generated previously [50] was used at a 1:10000 dilution in 5% BSA in PBS_T_. Secondary antibody goat anti-rabbit HRP (Thermofisher: 65-6120) was used at 1:20000 in 5% BSA in PBST. Western blots were developed using SuperSignal West Pico Plus (Thermofisher: 34580) and imaged using a BioRad ChemiDoc. Western blot analysis for quantification of relative protein levels was performed using ImageJ software.

### Growth assays

The growth assays + /- aTc were started by washing parasite cultures 3 times to ensure removal of aTc and then diluted to 1% parasitemia. A negative empty red blood cell 2% hematocrit control was prepared and treated in parallel. Flow cytometry samples were collected every 24 hours. Cultures were given fresh media every two days or split as indicated to maintain healthy cultures.

### Heat shock assay

A growth assay was set up and analyzed as described above; however, at the indicated timepoint cultures were moved to an incubator set to 41°C for 6 hours. Media was exchanged, and parasites were returned to 37°C for the remaining experiment.

### Cell cycle progression

To monitor lifecycle progression in tightly synchronized PfHSP40^KD^ parasites, cells were synchronized using a combination of sorbitol and percoll gradients. When parasites were approximately 8-hour rings, + /- aTc was started by washing parasite cultures three times to ensure removal of aTc, and then diluted to 2–3% parasitemia. Microscopy slides and flow cytometry samples were collected for multiple timepoints from the initial replication cycle all the way to the middle of cycle 3 to fully capture the cell cycle progression. Media was exchanged 10 times throughout the experiment to maintain healthy cultures.

### Flow cytometry

For flow cytometry samples, 50μL of cells were fixed and stored in 4% paraformaldehyde, 0.025% glutaraldehyde at 4°C. When ready to analyze, cells were washed with PBS and resuspended to 1% hematocrit in PBS. Then 50μL of cell suspension was diluted into 300μL of 0.3μg/mL acridine orange (Invitrogen: A3568) in PBS and analyzed by a Cytek Aurora flow cytometer. Gating and parasitemia was determined using FlowJo software gating for red blood cells (SSC-A vs FSC-A), single cells (SSC-A vs SSC-H), and then for infected red blood cells (B3-A vs B7-A). Cumulative parasitemia was determined by subtracting the uninfected red blood cell control collected at the same time, dividing each sample first timepoint parasitemia, and then multiplying by the split factor if cultures were split. To measure DNA content, median fluorescence intensity of the infected red blood cell population for the B3-A was used.

### Light microscopy

Microscopy slides of synchronized parasites blood smears were fixed 10 seconds in methanol throughout the asexual parasite lifecycle + /- aTc. All fixed slides were stained for 15 min with Giemsa stain (Sigma: SLCM6930) diluted 1:20 and imaged with an Olympus CX43 microscope and Olympus DL21 camera at 100X magnification. Images were cropped, adjusted for brightness, and exported using ImageJ.

### Fluorescence microscopy

Thin blood smears of late-stage PfHSP40^KD^ schizonts + /- aTc were prepared on Superfrost glass slides (Fisherbrand: 12-550-15) and air-dried. Smears were fixed in chilled methanol and stored at -20°C. During cycle 2, since PfHSP40^KD^ -aTc parasites lag in their progression, smears were made approximately 8 hours later than +aTc parasites. On the day of staining, the smears were air-dried and mounted with Prolong Gold Antifade with DAPI (Molecular probes: P36941). Images were captured using a Leica confocal DMi-8 microscope with a 40x/1.35 numerical aperture (NA) oil immersion objective. Serial z-sections of each image were gathered, and the z-stack with the best representation is illustrated in the figure. Images were analyzed by open-sourced ImageJ software. Approximately 30 individual schizonts in each parasite sample were scored using ImageJ thresholding combined with watershed and analyze particles.

### Drug sensitivity assays

All dose-response inhibition experiments were performed on at least three biological replicates of PfHSP40^KD^ parasites in technical duplicate + /- aTc. Parasite growth was measured on a CLARIOstarPlus (BMG LAB TECH) plate reader with Quant-iT PicoGreen dsDNA reagent (Invitrogen: P7581) staining. Data were fit to a non-linear regression to determine 50% inhibitory concentration (IC_50_) value using GraphPad Prism.

Dihydroartemisinin (DHA) and Bortezomib (BTZ) IC_50_ experiments were done with asynchronous PfHSP40^KD^ parasites on the same day as removing aTc or after 2 days without aTc. DHA (Caymen Chemicals: 19846) and BTZ (Caymen Chemicals: 10008822) were diluted in DMSO. For DHA two-fold dilutions from 0nM to 20nM and for BTZ, two-fold dilutions from 0nM to 400nM were added to 100μL of parasites and analyzed after 72 hours.

DHA ring-stage survival assays (RSA) were done with tightly synchronized 0–3-hour PfHSP40^KD^ rings after 2 days + /- aTc, adapted from methods as described [51]. Three-fold dilutions from 0nM to 1μM of DHA were added to 100μL of parasites for 6 hours. After the 6-hour DHA pulse, media was exchanged 4 times and cells were transferred to a new plate, maintained with or without aTc and analyzed after 72 hours.

Clofarabine IC_50_ experiments were done with synchronous 8-hour ring PfHSP40^KD^ parasites on the same day as removing aTc. Clofarabine (Sigma: C7495) was diluted in DMSO and added in 2-fold dilutions from 0μM to 1.6μM to 100μL of parasites analyzed after 96 hours.

### Proteomics

#### Sample preparation.

Proteomics samples were obtained by tightly synchronizing PfHSP40^KD^ parasites and washing off aTc as rings. During the cycle 1 and cycle 2 a sample was collected + /- aTc for 5 biological replicates when parasites were predominately trophozoite stage. For each replicate at the 2 separate time points, a 30mL 4% hematocrit sample with at least 5% parasitemia was collected. Samples were prepared by washing with PBS, adding 1% saponin to lyse red blood cells, then washing twice with cold PBS prior to flash freezing on dry ice. Parasite pellets were stored at -80°C until ready to run on LC-MS/MS by the CHOP Proteomics Core.

#### In-solution digestion.

Parasite pellets underwent lysis, solubilization, and digestion on an S-Trap (Protifi) following the manufacturer’s protocol [52]. Subsequently, the resulting peptides were de-salted using an Oasis HLB plate (Waters), dried via vacuum centrifugation, and reconstituted in 0.1% TFA containing iRT peptides (Biognosys Schlieren, Switzerland).

#### Mass spectrometry acquisition and data analysis.

Peptides were analyzed on a QExactive HF mass spectrometer coupled with an Ultimate 3000 nano UPLC system and an EasySpray source utilizing data independent acquisition (DIA). Raw data were searched using Spectronaut [53]. The MS2 intensity values for proteins generated by Spectronaut were used for bioinformatics analysis. Proteomics data processing and statistical analysis were conducted in R. The MS2 intensity values generated by Spectronaut were utilized for analyzing the entire proteome dataset. The data underwent log2 transformation and normalization by subtracting the median value for each sample. To ensure data integrity, we filtered it to retain only proteins with complete values in at least one treatment group (+ or - aTc). To compare proteomics data across groups, we employed a Limma (linear models for microarray data) t-test to identify proteins with differential abundance, with empirical Bayes smoothing and Benjamini-Hochberg method for multiple test correction. Lists of differentially abundant proteins were generated based on criteria of an adjusted P-value < 0.05 and absolute log2(fold change) > 1, resulting in a prioritized list for subsequent analysis.

#### Gene ontology and heat map.

Gene ontology (GO) analysis was done using ShinyGO, using the genes detected by proteomics as the background a false discovery rate cut-off of 0.05 and the GO Biological Process database [54]. For the heat map, Ward’s hierarchical clustering was done using Euclidean clustering distances for rows and columns. Data were processed and visualized using R studio.

## Supporting information

S1 Fig PfHSP40^KD^ parasites demonstrate a reversible knockdown phenotype. A) PCR tests confirm genomic integration of the TetR-DOZI cassette at the PfHSP40 locus in *P. falciparum.* The same primer set (indicated by black arrows) was used for PCR with 3D7 (WT) and PfHSP40^KD^ genomic DNA. Growth assays of asynchronous PfHSP40^KD^ parasites measuring cumulative parasitemia by flow cytometry every 24hrs cultured + /- aTc, adding back aTc on either B) day 2 or C) day 4 -aTc (indicated by red arrow). Parasites were split 1:6 after day 4. Data represents the mean + /- SEM of 3 biological replicates, missing error bars are too small to be visualized. Parametric unpaired t-tests between the add back aTc and -aTc condition were performed for the final day of collection (**p < 0.01, *** p < 0.001).
(TIF)

S2 Fig Heat shock after one day of PfHSP40 knockdown shows reduced parasite thermotolerance. A) Experimental design to assay thermotolerance: PfHSP40^KD^ parasites were subjected to a 6hr 41°C heat shock (HS) on day 1 + /- aTc. B) Anti-PfHSP40 immunoblot of PfHSP40^KD^ parasites + /- aTc in the control or HS condition collected immediately following HS. Blot is representative of 3 biological replicates. Parasitemia was measured by flow cytometry collecting every 24hrs following the HS for both. C) HSP40 expression on (+aTc) or D) off (-aTc). Cultures were split 1:4 after day 3 collection. Data represents the mean + /-SEM of 3 biological replicates, missing error bars are too small to visualize. Parametric unpaired t-tests were performed (*p < 0.05, **p < 0.01).
(TIF)

S3 Fig PfHSP40^KD^ parasites demonstrate a second cycle developmental defect. A) Tightly synchronized PfHSP40^KD^ parasites were monitored for lifecycle progression starting + /-aTc at 8hrs post invasion (HPI) through the third cycle of replication. During cycle 2, there is a developmental lag starting when +aTc is 84 HPI and continues as +aTc parasites enter cycle 3. Data is representative of 3 biological replicates. B) Histograms of infected red blood cell DNA content in PfHSP40^KD^ parasites + /- aTc from flow cytometry samples collected at time points indicated in part A. Starting at 84 HPI when the + aTc condition progresses into schizogony and increases the DNA content of cells, the -aTc condition lags. Entering cycle 3 at 102 HPI, the + aTc condition shows a large population with predominantly lower DNA content due to the newly invaded cycle 3 rings, while the -aTc has a smaller total population of cells with higher DNA content. Data is representative of 3 biological replicates.
(TIF)

S4 Fig Whole-Cell Proteomics of PfHSP40^KD^ parasites + /-aTc. A) Volcano plot of cycle 1 + /- aTc differential abundance analysis, PfHSP40 was the only protein with significantly different expression. B) Heat map of the normalized intensity of all 75 differentially expressed proteins cycle 2 + /- aTc across 5 biological replicates detected by proteomics. Hierarchical clustering was performed using Euclidean distance and Ward method for columns and rows. Peptides that were not detected are NA in grey.
(TIF)

S1 Table PfHSP40 Cycle 1 Proteomics. (XLSX)

S2 Table PfHSP40 Cycle 2 Proteomics. (XLSX)

S3 Table PfHSP40 Cycle 2 Downregulated GO Analysis. (XLSX)

S4 Table Primer List. (DOCX)

S1 Appendix PfHSP40 Gene Fragment Sequence. (DOCX)
