# Supplementary figures and images for: Essential role for PfHSP40 in asexual replication and thermotolerance of malaria parasites
Source: PLoS Pathog. 2025 Jul 8;21(7):e1013313. doi: 10.1371/journal.ppat.1013313 (PMC12258570; doi:10.1371/journal.ppat.1013313)

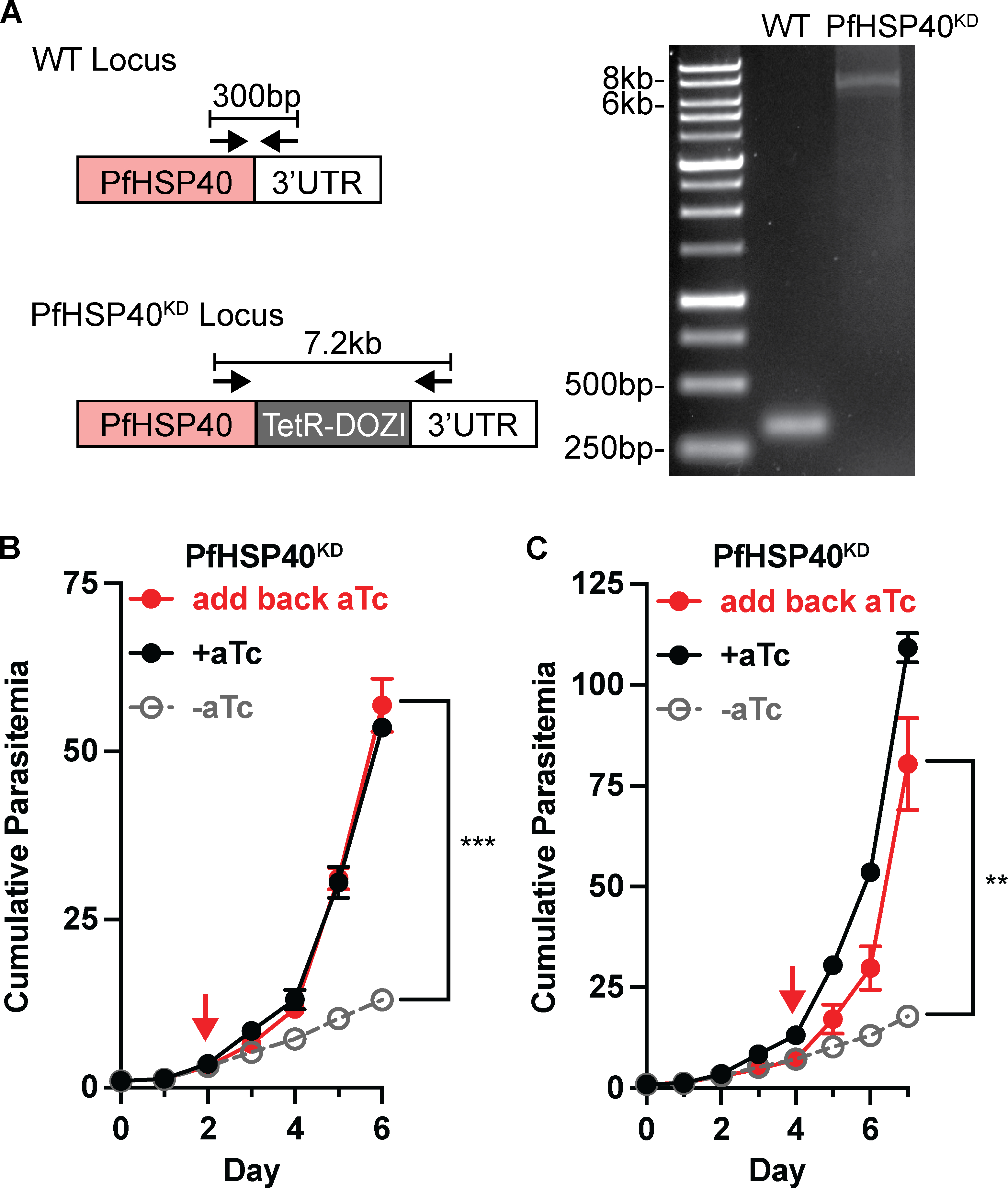

Supplement: S1 Fig — A) PCR tests confirm genomic integration of the TetR-DOZI cassette at the PfHSP40 locus in P. falciparum. The same primer set (indicated by black arrows) was used for PCR with 3D7 (WT) and PfHSP40KD genomic DNA. Growth assays of asynchronous PfHSP40KD parasites measuring cumulative parasitemia by flow cytometry every 24hrs cultured + /- aTc, adding back aTc on either B) day 2 or C) day 4 -aTc (indicated by red arrow). Parasites were split 1:6 after day 4. Data represents the mean + /- SEM of 3 biological replicates, missing error bars are too small to be visualized. Parametric unpaired t-tests between the add back aTc and -aTc condition were performed for the final day of collection (**p < 0.01, *** p < 0.001). (TIF) [file ppat.1013313.s001.tif]

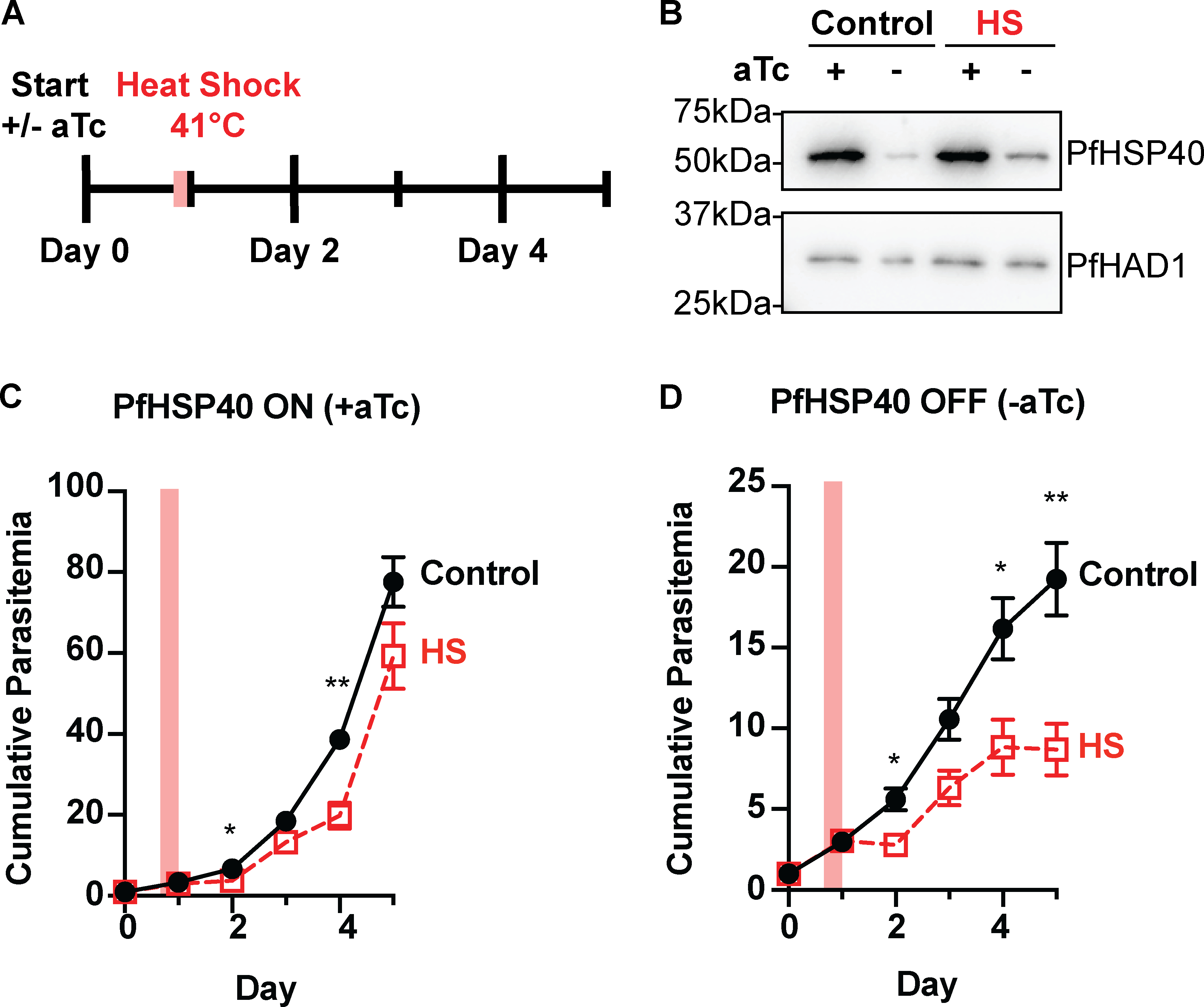

Supplement: S2 Fig — A) Experimental design to assay thermotolerance: PfHSP40KD parasites were subjected to a 6hr 41°C heat shock (HS) on day 1 + /- aTc. B) Anti-PfHSP40 immunoblot of PfHSP40KD parasites + /- aTc in the control or HS condition collected immediately following HS. Blot is representative of 3 biological replicates. Parasitemia was measured by flow cytometry collecting every 24hrs following the HS for both. C) HSP40 expression on (+aTc) or D) off (-aTc). Cultures were split 1:4 after day 3 collection. Data represents the mean + /-SEM of 3 biological replicates, missing error bars are too small to visualize. Parametric unpaired t-tests were performed (*p < 0.05, **p < 0.01). (TIF) [file ppat.1013313.s002.tif]

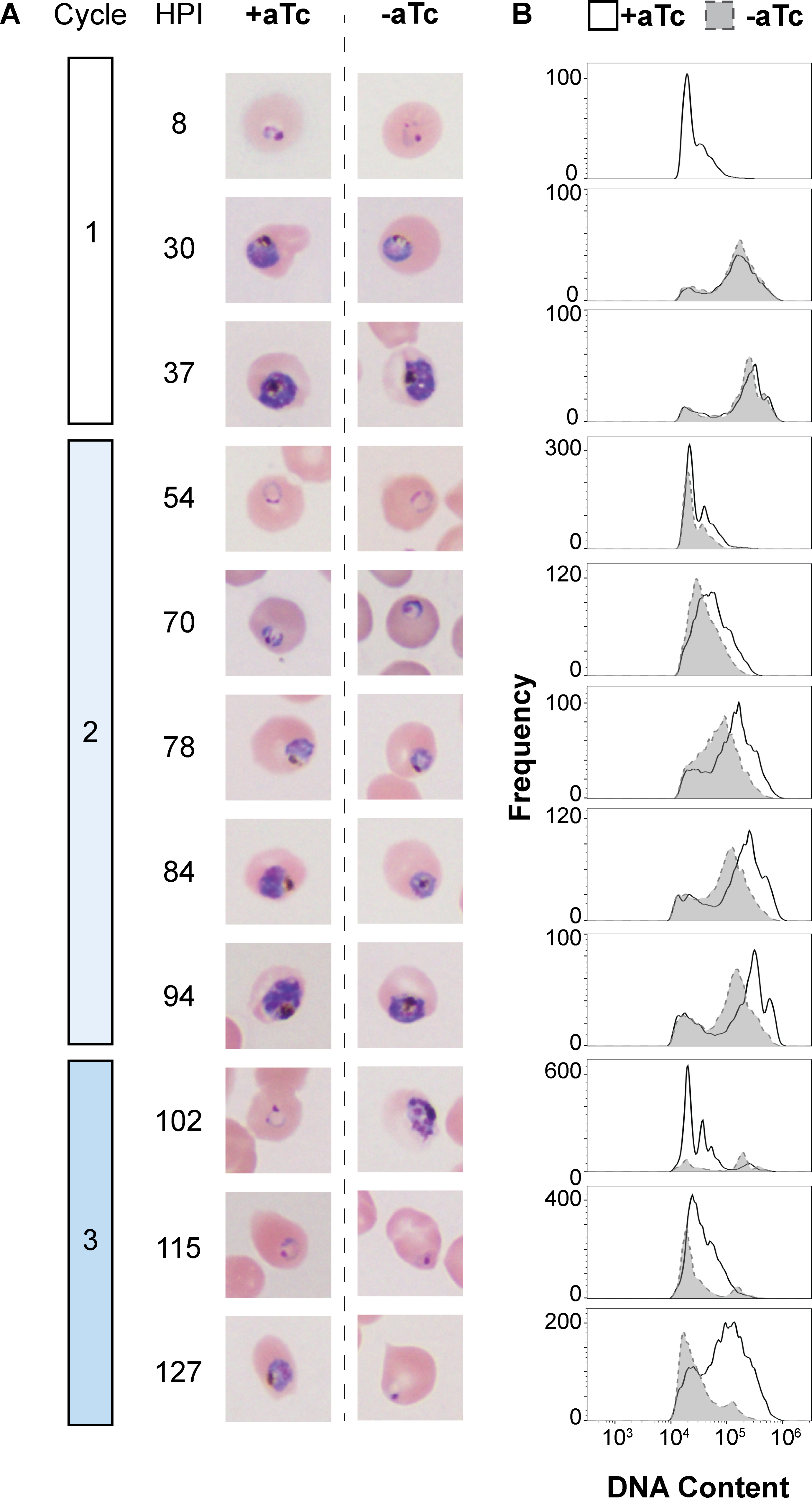

Supplement: S3 Fig — A) Tightly synchronized PfHSP40KD parasites were monitored for lifecycle progression starting + /-aTc at 8hrs post invasion (HPI) through the third cycle of replication. During cycle 2, there is a developmental lag starting when +aTc is 84 HPI and continues as +aTc parasites enter cycle 3. Data is representative of 3 biological replicates. B) Histograms of infected red blood cell DNA content in PfHSP40KD parasites + /- aTc from flow cytometry samples collected at time points indicated in part A. Starting at 84 HPI when the + aTc condition progresses into schizogony and increases the DNA content of cells, the -aTc condition lags. Entering cycle 3 at 102 HPI, the + aTc condition shows a large population with predominantly lower DNA content due to the newly invaded cycle 3 rings, while the -aTc has a smaller total population of cells with higher DNA content. Data is representative of 3 biological replicates. (TIF) [file ppat.1013313.s003.tif]

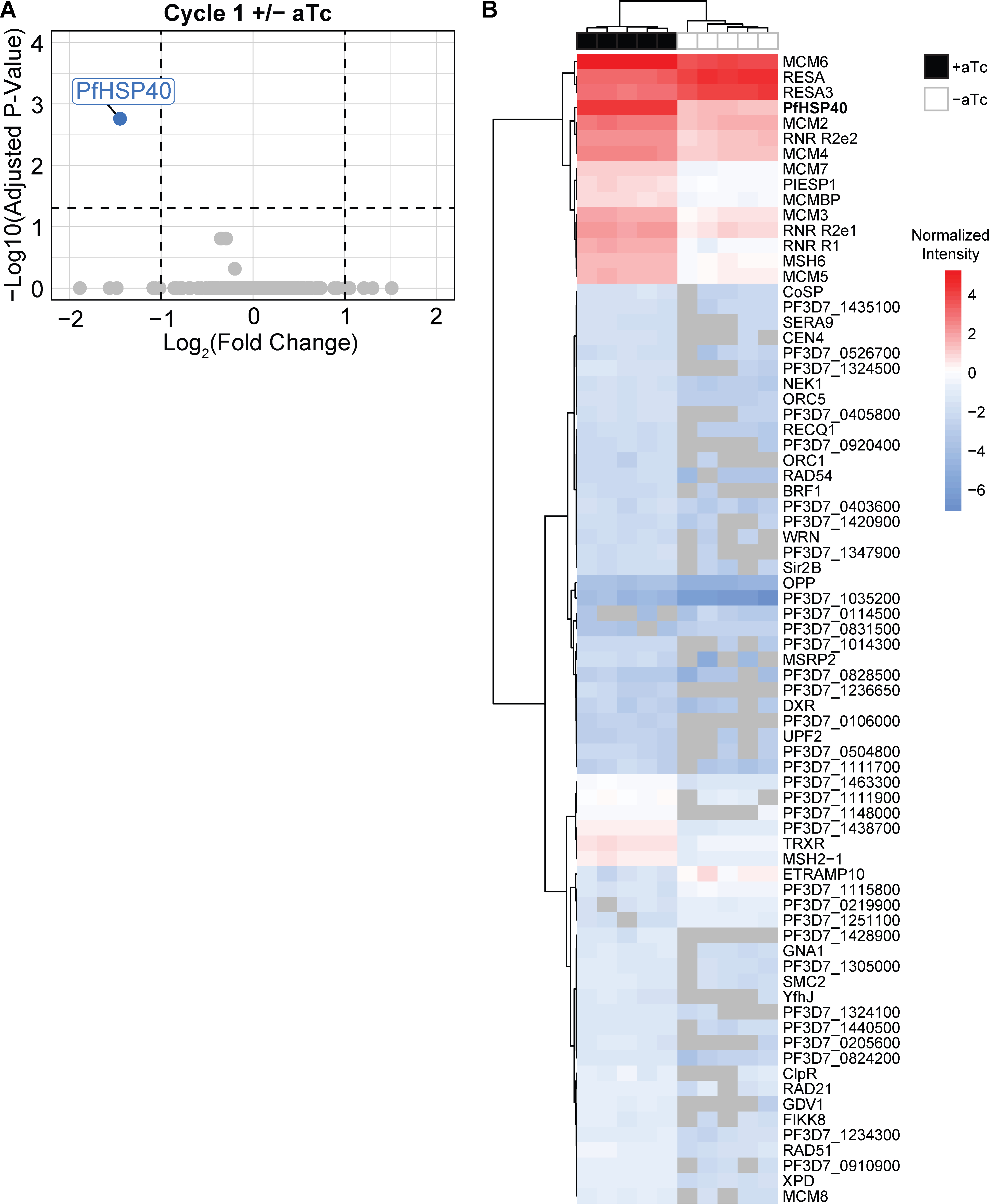

Supplement: S4 Fig — A) Volcano plot of cycle 1 + /- aTc differential abundance analysis, PfHSP40 was the only protein with significantly different expression. B) Heat map of the normalized intensity of all 75 differentially expressed proteins cycle 2 + /- aTc across 5 biological replicates detected by proteomics. Hierarchical clustering was performed using Euclidean distance and Ward method for columns and rows. Peptides that were not detected are NA in grey. (TIF) [file ppat.1013313.s004.tif]
