# Supplementary material for: Essential role for PfHSP40 in asexual replication and thermotolerance of malaria parasites
Source: PLoS Pathog. 2025 Jul 8;21(7):e1013313. doi: 10.1371/journal.ppat.1013313 (PMC12258570; doi:10.1371/journal.ppat.1013313)
Supplement: S4 Table — (DOCX) [file ppat.1013313.s008.docx]

| Primer A | PfHSP40 5’UTR F | GACTGTGCCGGCCGGAACATGCGTTCATCATAATGAATAAC |
| --- | --- | --- |
| Primer B | PfHSP40 5’UTR R | AATGACAAGGGCCGGTTATTTCTCGTGAATGTTCTACAAGA |
| Primer C | PfHSP40 3’UTR F | CTCGGATTACCCTGTTATTGTGTCTGTGAAATGAAATACG |
| Primer D | PfHSP40 3’UTR R | CTCGGATTACCCTGTTATTGTGTCTGTGAAATGAAATACG |
| Primer E | PfHSP40 Gene F | TCCAGGTCCAGCGATTCTCGGTAAAATGTACCCTTATGACG |
| Primer F | PfHSP40 Gene R | CGTCGTCTTTGTAGTCCCGTACGTTATTGTTGAGCACAAGC |
| Primer G | Sequencing Primer F | GGAAGGTGGAAGAGTAGCTTGT |
| Primer H | Sequencing Primer R | AATCCAGGTCCAATGACTTCGAAAGTTTATGATCC |
