# Supplementary material for: Essential role for PfHSP40 in asexual replication and thermotolerance of malaria parasites
Source: PLoS Pathog. 2025 Jul 8;21(7):e1013313. doi: 10.1371/journal.ppat.1013313 (PMC12258570; doi:10.1371/journal.ppat.1013313)
Supplement: S1 Appendix — (DOCX) [file ppat.1013313.s009.docx]

GCGATTCTCGGTAAAATGTACCCTTATGACGTTCCAGACTACGCTTATCCTTACGACGTCCCCGACTACGCGTATCCCTACGACGTTCCTGATTACGCTGCGATCGCAATGTTTTTCTCATCAGGTTTTCCATTTGACTCTATGGGTGGTCAACAAGCCCGTAGAAAAAGAGAAGTAAATAATAACAAATTTTACGAAGTTTTGAATTTAAAAAAAAATTGTACTACTGACGAAGTAAAAAAAGCGTATCGTAAGCTAGCTATTATTCATCATCCAGATAAAGGAGGTGATCCAGAAAAGTTCAAAGAAATATCAAGAGCATATGAAGTATTATCAGATGAAGAAAAAAGGAAATTGTATGATGAGTATGGTGAAGAAGGTTTAGAAAATGGAGAACAACCAGCTGATGCAACAGATTTATTTGACTTTATTTTAAATGCTGGAAAAGGAAAGAAAAAGAGAGGTGAAGATATTGTGAGTGAAGTAAAAGTAACACTTGAACAATTATATAATGGAGCAACAAAAAAGCTAGCTATAAGTAAAGATATTATATGTACAAATTGTGAAGGTCATGGTGGTCCAAAAGATGCTAAAGTAGATTGTAAACAGTGTAATGGAAGAGGAACAAAGACATACATGAGATATCATTCAAGCGTTTTACATCAAACTGAAGTAACTTGTAATACATGTCGTGGAAAAGGTAAAATATTTAATGAAAAAGATAAATGTGCTAATTGTAAAGGTATGTGTGTATTGAAAACACGTAAAATAATTGAAGTATATATTCCTAAAGGTGCACCAAATAAACACAAAATAGTTTTTAATGGTGAAGCTGATGAAAAACCAAATGTTATTACCGGAAATCTTGTTGTTATATTAAATGAAAAGCAACATCCTGTATTTAGAAGAGAAGGAATTGATTTATTTATGAATTACAAAATCTCTTTATATGAATCTTTAACAGGTTTTGTTGCAGAAGTTACACATCTTGATGAAAGGAAAATATTAGTAAACTGCACAAACAGTGGTTTTATAAGACATGGAGATATTAGAGAAGTTCTTGATGAAGGTATGCCAACATATAAAGATCCATTCAAAAAAGGAAATTTATATATTACATTTGAGGTTGAATATCCAATGGATTTAATTATAACAAATGAAAACAAAGAAGTTTTGAAAATCTTAAAAAAACAAAATGAAGTTGAAAAAAAATATGACTTAGAAAATAGTGAACTAGAAGTTGTTTCTTGCTCACCTGTTGATAAAGAATATATTAAAGTTAGAGTTACCAAACAACAACAACAACAACAACAAGAAGCATACGACGATGAAGATCATCAACCAGAAATGGAAGGTGGAAGAGTAGCTTGTGCTCAACAATAACGTACGG
